# A Survey of Software-Defined Networks-on-Chip: Motivations, Challenges and Opportunities

**DOI:** 10.3390/mi12020183

**Published:** 2021-02-12

**Authors:** Jose Ricardo Gomez-Rodriguez, Remberto Sandoval-Arechiga, Salvador Ibarra-Delgado, Viktor Ivan Rodriguez-Abdala, Jose Luis Vazquez-Avila, Ramon Parra-Michel

**Affiliations:** 1 Academic Unit of Electrical Engineering, Center of Research, Innovation and Development in Telecommunications (CIDTE), Autonomous University of Zacatecas, Zacatecas 98000, Mexico; jrgrodri@uaz.edu.mx (J.R.G.-R.); sibarra@uaz.edu.mx (S.I.-D.); abdala@uaz.edu.mx (V.I.R.-A.); 2 Department of Electronic and Computer Engineering, University of Cordoba, 14071 Córdoba, Spain; 3 Facultad de Ingeniería, Universidad Autónoma del Carmen, Carmen 24180, Mexico; jvazquez@pampano.unacar.mx; 4 Department of Electrical Engineering, Communications Section, CINVESTAV-IPN, Guadalajara, Jalisco 45019, Mexico; rparra@gdl.cinvestav.mx

**Keywords:** Networks-on-Chip, challenges, opportunities, Software-Defined Networks-on-Chip, survey

## Abstract

Current computing platforms encourage the integration of thousands of processing cores, and their interconnections, into a single chip. Mobile smartphones, IoT, embedded devices, desktops, and data centers use Many-Core Systems-on-Chip (SoCs) to exploit their compute power and parallelism to meet the dynamic workload requirements. Networks-on-Chip (NoCs) lead to scalable connectivity for diverse applications with distinct traffic patterns and data dependencies. However, when the system executes various applications in traditional NoCs—optimized and fixed at synthesis time—the interconnection nonconformity with the different applications’ requirements generates limitations in the performance. In the literature, NoC designs embraced the Software-Defined Networking (SDN) strategy to evolve into an adaptable interconnection solution for future chips. However, the works surveyed implement a partial Software-Defined Network-on-Chip (SDNoC) approach, leaving aside the SDN layered architecture that brings interoperability in conventional networking. This paper explores the SDNoC literature and classifies it regarding the desired SDN features that each work presents. Then, we described the challenges and opportunities detected from the literature survey. Moreover, we explain the motivation for an SDNoC approach, and we expose both SDN and SDNoC concepts and architectures. We observe that works in the literature employed an uncomplete layered SDNoC approach. This fact creates various fertile areas in the SDNoC architecture where researchers may contribute to Many-Core SoCs designs.

## 1. Introduction

Current Many-Core System-on-Chip designs exploit the computation power and parallelism available for dynamic workload systems such as mobile smartphones, IoT, embedded devices, desktop, and data centers. A Many-Core System-on-Chip has a number from tens to thousands of processing cores and memories interconnected by an on-Chip network. The processing cores vary from CPU, GPU, Intellectual Property (IP), programmable hardware, and specialized neuromorphic hardware for artificial intelligence, among many others.

NoCs attracted the attention of the most important companies in the Silicon Valley industry. Several large companies purchased NoC companies, as shown in Table 1. This trend indicates the importance of accelerating new chips’ design by using an NoC solution from companies that have proven their products in the marketplace. Arteris is currently the only significant player in the NoC scene; however, this leaves a fertile path for innovation and creating solutions for recent market niches.

An NoC provides the means to meet functional, power, and cost requirements; and achieve operational safety and data reliability requirements when required. The development of chips for current applications demands that the interconnection networks inside and outside the chip have adequate bandwidths to avoid causing bottlenecks that degrade the processing. Table 2 shows some chip developments by the most relevant electronics industry companies. Some of these developments reach the tens of thousands of processing cores that result in fundamental challenges to the interconnection on a chip [4,5]. It is important to note that all companies employ an on-Chip network to connect the processing cores. This fact highlights the importance of NoCs in the industry.

As the number of processing elements and memory in an SoC increases, the elements’ interconnection becomes the critical element in the design process. SoC architectures evolved with more types and numbers of hardware accelerators added with each new version of a chip. Current data processing techniques require hardware customization for algorithm acceleration but also dataflow optimization. An NoC allows designers to separate computational problems from communication problems in a Many-Core SoC. Thus, an adaptable NoC is crucial for the system performance. Having a scalable network of thousands of processing cores is not an easy task, and a layered approach is needed to address different problems in a modular way. However, The NoC design complexity increases with the adaptive requirements due to dynamic workload [16], which makes the management of a network of thousands of elements a complex and interesting problem.

In the Many-Core arena, the NoC becomes the performance bottleneck and introduces the need for network management. Network management problems also occur in traditional networks with hundreds of devices connected, for example, a data center or a university campus. Software-Defined Networking (SDN) has been a paradigm shift that elegantly solves computer networks’ management problems. By separating the control logic from the data transmission logic, it simplifies the network device implementation. It logically centralizes decisions in a controller or Network Operating System with a global vision of the network. It can perform decisions or make optimizations with more and better information to achieve system performance goals. SDN’s main benefit is to consolidate and simplify the network devices’ management of different vendors employing an SDN controller [17].

The SDN concept copes with the management problem in Many-Core systems based on NoCs. A Software-Defined Network-on-Chip (SDNoC) brings benefits such as higher flexibility for runtime and self-adaptive network management, and a reduced hardware complexity for routers. An external software entity, denominated SDNoC controller, carries features such as Quality of Service (QoS), fault tolerance, and power management, yielding simpler NoC routers. Therefore, the routers are configurable, and their primary function is to redirect NoC packets according to the SDNoC controller policies and rules. The SDNoC controller has a global view of the NoC and its resources. Thus, the SDNoC controller globally optimizes the networks with different goals such as fault mitigation, load balance, power consumption, and QoS for real-time flows. Furthermore, the controller can combine several goals to achieve multi-objective management [17].

The SDNoC approach bears a simpler and configurable router architecture with an external software controller that changes the routing paths according to real-time constraints, which differs from other software-based control proposed in the literature [17,18,19]. In 2015, we introduced a layered SDN architecture in the NoCs, employing the SDNoC concept [20]. This architecture allowed to simplify the designs of the system’s interconnections. Several works implemented the SDNoC approach, but they lack a clear layer definition. The layered architecture’s original vision thrives as an open platform for innovation where the designer reuses SDNoC solutions, improves time to market, and reduce non-recurring engineering cost. However, the proposal was not fully understood, and SDNoC research took different ways.

This paper contributes with a survey of the literature related to SDNoC. We explain the motivation for an SDNoC approach and clarify both SDN and SDNoC concepts. Moreover, it shows how state-of-the-art has overlooked some details of an SDN architecture and presents some research directions to correct the way to a complete architecture for SDNoC-based systems.

We present the rest of this work as follows: Section 2 explains the motivation for a SDNoC approach. We describe the SDN concept in Section 3 and clarify the SDNoC architecture in Section 4. Section 5 surveys the works related to Software-Defined Networks-on-Chip and classifies the literature regarding the SDN features. Finally, Section 6 presents the challenges and the opportunities revealed in the literature survey, and Section 7 integrates the conclusions and perspectives.

## 2. The Motivation for an SDNoC Approach

The most uncomprehended aspects of Software-Defined Networks-on-Chip are the motivation and the management problems associated with Many-Core systems that it solves. In this section, we present the NoC basics to understand the NoC management issues. We also discuss network management optimization problems related to increasing the number of processing elements in an NoC-based system.

### 2.1. NoC Basics

**Figure 1:**
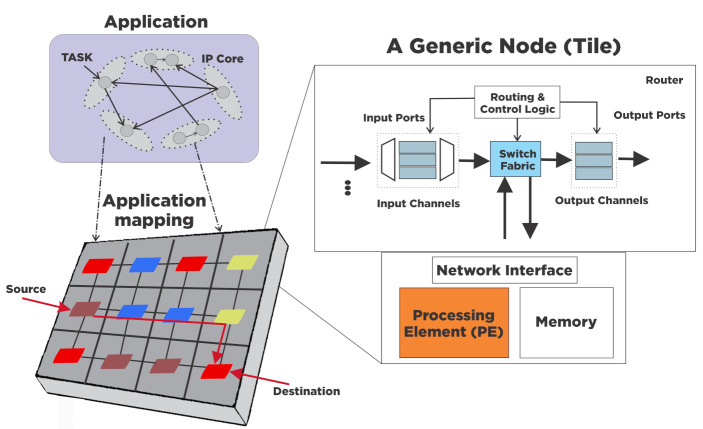
NoC architecture, application, and its mapping to the NoC. The micro-architecture of a node, which consists of an on-chip router, buffers, and processing element (PE), is also shown on the right-hand side of the figure [22].

In the NoC-based design, several processes and techniques from the classic computer networking approach are introduced into SoC design [21]. The NoC concept employs a packet-switching fabric for on-chip communication. In this architecture, the SoC’s computing power is divided into processing tiles, each tile formed by processing elements, memories, Network Interfaces (NI), and routers, as Figure 1 shows. Network Interfaces decouples computation and communication domains. At the source, NI converts the data from the processing cores into packets. NI merges all the packets to gather the message and delivers it to the destination’s processing core. The central NoC elements are routers and links. Routers move packets from source to destination nodes, and point-to-point links connect two neighboring routers.

In the following section, we describe the design issues and goals related to an NoC-based SoC design.

### 2.2. Design Goals

The NoC concept permits designers to decouple the communication fabric from the processing and storage elements [23]. Thus, designers optimize the interconnection infrastructure independently of the functionality. With the use of models and techniques from networking and parallel processing, designers can view a complex SoC as a micro-network of multiple blocks [21]. Furthermore, abstraction levels facilitate a modular design and encourage innovation in every layer guaranteeing separation of concerns.

However, the design process has optimization goals with one or several metrics involved, which may complicate the design space exploration. In the following, we present the metrics most used in the literature.

#### 2.2.1. Performance and QoS

It is crucial to evaluate NoC designs in terms of their performance metrics, regardless of their implementation [23]. Although performance analysis can include several metrics such as reliability and jitter, we focus on the most used metrics in the literature, throughput, latency, and QoS.

**Throughput** is the rate at which packets are delivered by the network, with units of bits per clock cycle, we avoid using packets/cycle or flits/cycle since the packet size or flit size may change from one implementation to another. We measure the throughput by counting the packets that arrive at destinations over a time interval for each source-destination pair and computing the rates [24]. Additionally, we can define the throughput as the maximum load the network can physically handle [23].

**Latency** is the time in clock cycles that a packet needs to traverse the network from source to destination [24]. We define transport latency as the time (in clock cycles) that elapses between a message injection into the network at the source node, and the end of packet reception at the destination node [23].

**Quality of Service** encloses a collection of design requirements in specific performance metrics in a differentiated services scheme. Hence, the associated metrics to one application or source-destination pair must fulfill a certain performance level, for example, a maximum latency value [23].

#### 2.2.2. Reliability and Fault Tolerance

Although current technology permits us to incorporate thousands of processing cores due to the shrinking transistor geometries and rigorous voltage scaling, it brings some reliability issues. The vulnerability of fabricated NoC components affect their reliability, and it is due to transient and permanent faults, as shown in Table 3. Thus, a significant challenge is to enhance the system reliability [25].

Reliable execution of applications even with one or more faults is a desirable capability on NoC-based systems. A network degrades gracefully if it gradually reduces its performance with the number of faults. This ability is called fault tolerance [24], and we can measure it as the number of faults it can withstand before breakdown.

#### 2.2.3. Thermal Management

Many-core NoC-based designs need to cope with the high power density and temperature effects in delay, leakages, and reliability [28]. Thermal hotspots cause timing issues that force designers to opt for wider timing margins that degrade performance. The rise in on-chip components’ temperature may lead to permanent failures and degrade system reliability [29]. These facts make the thermal problem a vital challenge for Many-Core systems.

Throttling is one technique to reduce network temperature by lowering the clock frequency of some network components [28]. Routed traffic is directly related to switching frequency, power dissipation, and temperature increase. The less traffic the router processes, the lower its temperature is. This method effectively decreases the power generation in a network section. However, if we apply the throttling strategy until the router is off by power gating, we disconnect the router from its neighbors. In consequence, we changed the topology, affected the routing process, and degraded the NoC performance.

#### 2.2.4. Power Efficiency

Power efficiency is the ultimate challenge for chip designers, from battery-operated embedded SoCs to datacenter-specialized processing chips. As we mentioned before, thousands of cores and their interconnection must coexist in a single chip in the Many-core era. Transistor power leakage thrives its power consumption. Consequently, we must control the chip temperature to satisfy the manufactured maximum temperature, to avoid permanent chip damage [30]. The NoC consumes a significant percentage of the chip’s energy; then, energy efficiency is a crucial design goal in NoC-based systems.

Dark (power gating) silicon and dim (dynamic voltage/frequency scaling) silicon are two techniques to achieve energy efficiency in Many-Core chips [30,31,32]. Since power depends on voltage and frequency directly, the NoC must dynamically configure links and nodes depending on the traffic demands to extend chip power and thermal budgets, by scaling node-voltage and link-width, or power gating. Reza et al. affirm that 21% and 50% of chip resources may have to be dark at 22 nm and 8 nm process technology, respectively [30].

#### 2.2.5. Security

The Internet of Things increases Internet connectivity from the data center to the battery-powered NoC-based end devices. However, security vulnerabilities came with connectivity, which conveys that IoT devices represent a risk to any system. These systems download software code and firmware updates through their Internet connection. However, the downloaded software may be malicious and modify the system operation, retrieve secret information, or disrupt their services. Since the NoC is a shared medium is the preferred center of hackers’ attacks, it will affect computation and communication services [33]. In the IoT context, NoC-based systems integrate cryptographic hardware core for confidentiality and authentication security services. These components are prone to side-channel attacks. The cryptographic core’s measurements of the execution time, power consumption, and electromagnetic (EM) radiation are classical side channels attack. Attackers optimize cache attacks by detecting NoC communication patterns of sensitive traffic [34]. Thus, the attacker may compromise the complete embedded system’s security by the collision of malicious and sensitive traffic in the NoC.

### 2.3. NoC Optimization Problems

In this work, we focus on NoC management problems, and we leave aside programming and logic implementation issues to focus on the communication processes optimizations. Therefore, we present the most used design strategies applied to NoC management.

The building process of a suitable topology is called **topology design**. Tasks constitute applications, and the design process allocates every task into one processing element, see Figure 1, this process is called **application/task mapping**. Then, we defined the time of execution of every task, according to some criteria; this is called **task scheduling**. Tasks have communication requirements expressed as data dependencies among pairs of processing elements. According to some criteria, the path definition from a source-destination communication pair is called **routing**. All the processes mentioned above are interrelated, causing changes produced by one process to affect the others [35].

#### 2.3.1. Topology Design

An NoC topology is a description of each router’s connection to its neighbors. Some of the well-known topologies of NoC are Mesh, Ring, Torus, Star, Butterfly, and Fat Tree. Topology affects latency, throughput, area, fault tolerance, and power consumption [35]. The suitable NoC topology should have the following features: large bisection bandwidth, low node degree, small diameter, and low average distance [25]. Due to its large bisection bandwidth, simple node connection, and low complexity routing algorithm, the mesh is the most popular topology. However, it is important to design a topology that adapts to applications requirements, i.e., an application-specific topology [36,37]. Dynamically reconfigurable hardware such as FPGA technology enables adaptable topology.

#### 2.3.2. Scheduling

Another important strategy in NoC design is communication and task scheduling. The NoC-specific issue to cope in scheduling is to take into consideration the complex effects of the network (e.g., congestion), which may change dynamically during task execution [22].

#### 2.3.3. Mapping

The mapping process assigns the selected processing elements or cores into a topology, i.e., the places described by the topology nodes [22]. Once we selected a particular topology and application tasks assigned and scheduled into processing cores, the mapping determines each processing element’s neighbors, impacting both performance and energy consumption, and defines possible thermal hotspots [31].

#### 2.3.4. Routing

The routing process defines the route(s) or path(s) that a message takes from every source-destination pair of processing elements [22] and affects all the goals in the NoC design. The hop count or latency is directly affected by the route taken. Throughput is affected by congestion, which depends on the capacity of the routing to load balance. Each hop translates into a router energy overhead increasing power dissipation. Finally, routing influences reliability as it chooses routes that avoid faults [22].

#### 2.3.5. Management

The increasing number of processing cores in the current NoC-based system imposes new management and supervision requirements [18]. Some processing elements in the network serve as controller cores, which keep track of the status of the system and provide this information to the rest of the processing elements [18].

Some works described the concept of a cognitive network that can observe, act, learn, and optimize its performance [38,39]. This concept imposes management and supervision requirements on NoC-based systems. However, a modular and widespread management framework for Many-Core NoCs is still missing from the literature.

### 2.4. Summary

We have several possible solutions, which form the design space, for each of the above goals using different design strategies. To find the optimal solution for the design problems, we must explore the design space. Exhaustively searching the solution space becomes almost impossible since all the above optimization problems are NP-hard problems. Then, the literature envisaged techniques based on heuristics, meta-heuristics, and evolutionary approaches to explore the design space [25]. Table 4 shows some works in the literature that solve some of the issues described above. However, these solutions are not reusable or interchangeable, which increases the time to market and the non-recurring engineering cost. We believe that an SDNoC approach will benefit developing a system with optimizations engines based on the work classified in Table 4. In the sections below, we explain the details of the systems architecture to achieve this goal.

As Grecu et al. state, the NoC paradigm success relies on the standardization of the interfaces [23], here we focused on the management and control interfaces.

## 3. Software-Defined Networking Concept

In a conventional networking context, a simple network management architecture has three planes: data forwarding, control, and management. (1) Data forwarding corresponds to the network devices such as routers, switches, and hubs that transmit data from source to destination. (2) The control plane corresponds to the forwarding decisions, traditionally implemented in a distributed manner within the data plane elements, and protocols associated with the information exchange for the decision process. (3) The management plane includes the software services used to monitor and configure in situ or remotely the control functionality.

### 3.1. SDN Motivation

Several problems push the industry to a new networking paradigm, in which administrators may create an open and programmable network [58]. In a traditional networking environment, vendors bundled control and data plane inside of forwarding devices. Every device has its low-level configuration procedure defined by its manufacturer [59]. Furthermore, several instances of the same machine with different software versions may coexist within a network. This fact complicates the (re)configuration process when the administrator copes with faults or a load change in the network. Sometimes the configuration process is manual labor in every forwarding device in the network. The larger the network is, the more challenging it is to spread network policies on the entire network devices simultaneously. Moreover, forwarding devices may have different features or capabilities. Therefore, some configurations could leave out portions of the network with old or limited devices. The managing and control of the network become severe problems for the network administrators.

### 3.2. SDN Definition

**Figure 2:**
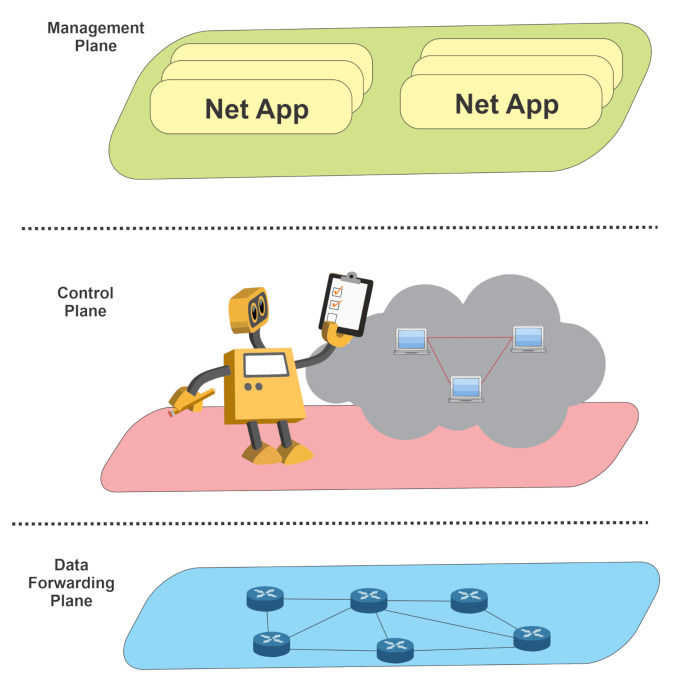
Software-Defined Network architecture presents three planes: data forwarding, control, and management.

Software-Defined Networking separates the network’s control logic from forwarding devices. Then forwarding devices became more uncomplicated, and an external SDN controller implements the control logic. SDN controllers have mechanisms to disseminate configurations through the network. SDN is a networking approach that defines a layered architecture for control and management, similarly to the OSI or TCP/IP layered architecture for data. As Figure 2 shows, an SDN network has three vertically integrated parts: management, control, and data planes. Such layered network architecture through a separation of concerns allows control and management solutions with a modular approach. SDN is a popular solution to manage large and complex networks in the networking industry [60] that changes the limitation of traditional network infrastructure [59].

An SDN network behaves as follows. The applications use the services provided by the management plane to establish network requirements. The management plane has network orchestration functions such as load balancing or fault detection, which take these requirements to generate policies. The control plane takes these policies and configures every network device in the data plane, consisting of every forwarding device in the network [60].

### 3.3. SDN Origins

Nick McKeown et al. initiated the SDN revolution at Stanford University in 2008 to satisfy the need for a better approach to managing networks on a campus [61]. They set goals to experiment with new protocols, an open network architecture, and a programmable network. One of the factors that inspire the SDN initiative was the burdensome network operation derived from every vendor’s configuration procedure. In [62], Gude et al. proposed a Network Operating System (NOS) to control, configure, and manage network devices. The NOS generates a software abstraction for every networking device in the network. The software abstraction builds a complete view of the network in which optimization routines generate networking device configurations adapted to the application requirements. In 2009, McKeown presented the Software-Defined Networking approach formally to satisfy the programmable network requirement [58]. He defined a programmable, reconfigurable, adaptable, and software-defined forwarding infrastructure with standard, reusable, and well-defined programming and communication interfaces that were hardware-independent and vendor-independent.

### 3.4. SDN Features

In computer networks, the term Software-Defined Networking (SDN) refers to a network architecture where the control plane resides outside from the forwarding devices. However, the network industry often has referred to anything that involves software as being SDN. In this paper, we follow the approach taken by [59,63], where SDN comprehend an architecture with the following features:

1. Network devices are simple forwarding elements (packet retransmission); SDN separates control functionality (route computation) from forwarding.
2. The network makes forwarding decisions based on flows instead of destination and source addresses. We define flow as a set of packets in which field values match some predefined criteria, such as a source-destination pair.
3. It generates a software abstraction of network logic and a global view of the network. SDN moved the control logic to an external entity, an SDN controller, or a Network Operating System (NOS). A NOS generates a consistent and centralized view of the network in which network applications run.
4. Networks achieve their programmability through software applications running on top of the NOS that interacts with the underlying data plane devices.
5. SDN has Software/Hardware interfaces to program the network, which permits adaptation of forwarding devices to applications running above.

With the principles mentioned above, we propose a Software-Defined Network-on-Chip (SDNoC) Architecture [20,47]. However, it is worth noting that on-Chip networks have different requirements than computer networks, for example, power, throughput, and delay. Nevertheless, they still share some basic principles as switching, routing, arbitration, among others. This paper aims to improve this vision with a clearer and precise explanation, which may accelerate the design, implementation, performance, management, and reconfiguration of NoC-based systems through well-defined services and interfaces between abstraction layers.

## 4. The SDNoC Layered Architecture

**Figure 3:**
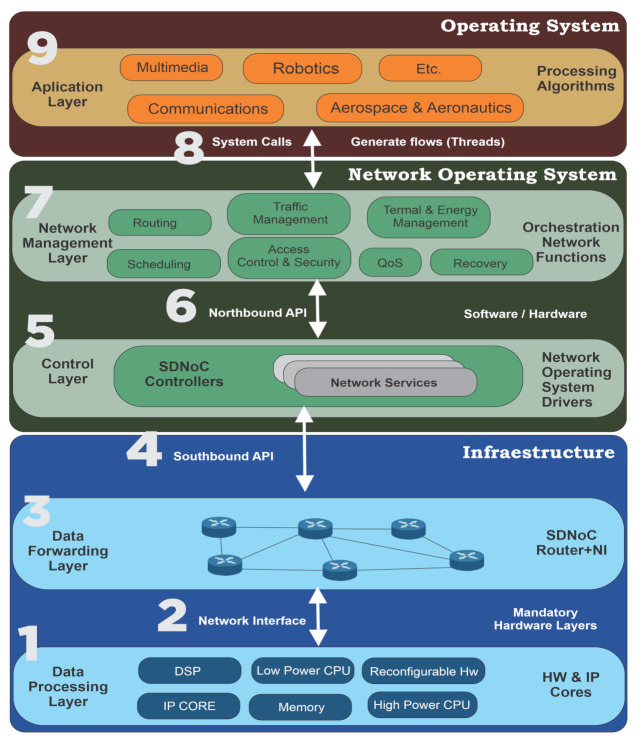
Software-Defined Network-on-Chip Architecture. Odd numbers represent the layers, and even numbers represent the HW/SW interfaces. A different color represents each segment.

As we pointed before, an SDN architecture focus on the administration and operation processes of the network. In a Many-Core NoC, these processes will become of great importance as the power and performance requirements become more difficult as the number of processing elements increase. The proposed SDNoC architecture presents three segments, Applications, Network Operating System and Infrastructure; and five layers: Applications, Network Management, Control, Data Forwarding and Data Processing, as shown in Figure 3. The main difference between our architecture and computer SDN is that we included a data processing plane. Every layer brings service to an upper layer through a well-defined interface. In the following, we describe every domain and layer in a bottom-up manner.

### 4.1. Infrastructure Segment

The infrastructure segment is responsible for the functions of forwarding and processing data. It covers data forwarding and data processing layers (1 and 3, respectively in Figure 3). Due to its operation, it is a mandatory hardware layer. The implementation can vary from manufacturer, designer, application, customer, or budget. The only imperative requirement for this layer is the southbound interface to communicate with the NOS segment.

#### 4.1.1. Data Processing Layer

It consists of the NoC’s processing elements such as CPUs, IP cores, Memories, reconfigurable hardware, or Direct Memory Access (DMA). These elements implement the basic functions of the algorithms in the SoC’s applications. A Network Interface connects the processing elements with the NoC. The services offered by this layer are:

1. General data processing (on CPUs);
2. Storage of data for further processing (Memories);
3. Specialized data processing (on IP cores or reconfigurable Hardware);
4. Improve data access latency (through DMAs).

This layer is present in every SoC design, whether it uses an NoC or not.

#### 4.1.2. Network Interface

The Network Interface generates data packets/transactions according to the NoC’s protocol, shown as (2) in Figure 3. It adapts data from the processing elements to the network and vice versa. Due to the data forwarding element’s buffers available, the packets/transactions size may have a maximum limit. The Network Interface split the messages into several packets/transactions and verifies that the messages arrive correctly at the destination.

#### 4.1.3. Data Forwarding Layer

It consists of routers, Network Interfaces (NI), switches, and buses that integrate the on-Chip network’s interconnection. Its main function is the transmission of data from its source to destination. In this plane, the upper layer interface is called the southbound interface in the SDN argot. The services offered by this plane are:

1. Send data from source to destination in a packet-based way (normal mode);
2. Send data from source to destination in a flow-based way (SDN mode);
3. Execute re/configuration in the forwarding devices (for each link or router);
4. Collect the state and statistics of the forwarding devices (for each link or router).

The Data forwarding layer’s elements in an SDNoC architecture are programmable components. A bus element could change its access priorities in a programmable manner; in a switch, we could program the output port selection; or a router’s next hop in a path to the destination. Traditional NoC-based systems have the same network components without the programmability or (re)configuration capabilities.

#### 4.1.4. Southbound Interface

The Southbound Interface, shown as (4) in Figure 3, communicates the data plane in the data forwarding layer in the infrastructure segment with the control layer’s control plane at the Network Operating System segment. This interface uses a protocol such as [17,64] to establish a secure channel. Both layers (Data Forwarding and Control layers) must implement this protocol. Its purpose is to communicate the controllers’ configurations to the forwarding devices’ statistics and states’ forwarding devices. In this way, a controller can adapt the NoC to an application or a particular situation. For example, a fault or an energy-saving state. Moreover, the controller can monitor the network elements and generate a general condition of the network. As we will state in the following sections, most of the SDNoC implementations reported in the literature missed this interface.

### 4.2. Network Operating Segment

The Network Operating System segment configures the infrastructure segment with policies generated from the application segment’s requirements. Moreover, this segment monitors the NoC’s elements and build a complete view of the NoC’s state. The Network Operating System possesses two layers: Control and Network Management, showed as (5) and (6), respectively in Figure 3.

#### 4.2.1. Control Layer

The control layer determines the mechanisms which allow the (re)configuration of the data forwarding devices. It comprises the SDN controllers, which establish a packet or flow-based connection with the southbound interface’s data forwarding device. Once they establish a secure connection, SDN controllers can send the configuration and collect the state and statistics data from the forwarding devices. An NoC can have one or several SDN controllers depending on the applications’ network size, traffic, and complexity. Each SDN controller in the NoC can have a partial network view, but once combined, the control layer can produce a global network view. We can categorize the SDN controllers by the forwarding device they control—router or switch-based, the switching mode such as circuits or packets, to mention a few. Then, we add a driver in the Network Operating System implementations for each kind of SDN controller. The control layer communicates with the upper layer using the Northbound interface. Also, the control layer offers the following services:

1. Send the configuration to a specific set of nodes in the network;
2. Collect state and statistics’ data from a specific set of nodes in the network;
3. Generate a global or partial view (state) of the network.

This layer is present in most of the SDNoC works in the literature but with limited functionality.

#### 4.2.2. Northbound Interface

The Northbound Interface, shown as (6) in Figure 3, communicates the Control layer with the Management layer in the Network Operating System segment. Both layers (Control and Management layers) must implement this protocol. Its purpose is to communicate the network optimization engine’s result’ to the controllers and the network’s state from the controllers. In this way, a network optimization engine can (re)adapt the NoC to the applications or particular situations. For example, fault detection, energy-saving states, or new applications. As we will state in the following sections, most of the SDNoC implementations reported in the literature missed this interface.

#### 4.2.3. Network Management Layer

The network management layer consists of several optimization engines that modify the network functions such as routing, scheduling, traffic management, access control and security, Quality of Service, thermal and energy management, and recovery. Such engines, called orchestration network functions, rely on a partial or global view of the network, which is proportioned by the control layer. Such functions allow programming of the NoC while maintaining several QoS requirements in throughput, delay, and power. The network management layer orchestrates the required combination of the optimization engines and organizes applications in the network. Moreover, the network management layer generates policies and configurations passed to the control layer via the Northbound interface to adapt the data forwarding plane to the applications. The optimization engines are modular; then, if a feature is not present or has to be added/removed, the change has no impact on the other engines. This layer interacts with the applications through the network operating system’s calls that generate threads or flows in the network. This layer offers the following services:

1. Optimization of an application’s mapping in terms of the engines available, e.g., energy or QoS;
2. Orchestration of the network functions to optimize the NoC globally;
3. Orchestration of the applications running in the NoC;
4. Mapping applications to the network (in conjunction with the NOS’s calls);
5. Dynamic or static resource allocation in the NoC and network abstraction generation.

#### 4.2.4. NOS System Calls

The NOS system offers its services to applications through system functions, shown as (8) or system calls in Figure 3. The applications call –in a software context– these system functions to access these services. Both NOS and applications need to implement these interfaces. Applications call the functions, and NOS’s implement the functions within their code. As we add services in new versions of the NOS, system functions can be added and called by applications.

### 4.3. Applications Segment

Finally, the application segment is where the applications reside, shown as (9) in Figure 3. Traditionally designers implement this over an NoC-based SoC platform.

#### Applications Layer

This layer holds the different applications running in the NoC. Each application or processing algorithm introduces a communication graph where the nodes are the functions of the processing elements in the NoC, and the edges are the data dependencies among them. The weights of the graph’s edges are the average Packet Injection Rates (PIRs) for the traffic between two nodes. Then, every application generates its graph and requirements and exhibits them to the Network Operating System. The Network Operating System will orchestrate the network functions to guarantee the requirements of the application requirements and the operational budget of the NoC, e.g., power and thermal restrictions.

The list of services presented here for all the layers is by no means complete; as the architecture matures, more services can be added, replaced, or eliminated. It is important to note that we present this architecture from the network management point of view, i.e., we only considered the control and administration of the NoC. The data communications can follow an OSI reference model, not exposed here to avoid confusing the reader and for space issues.

### 4.4. SDNoC Implementations

**Figure 4:**
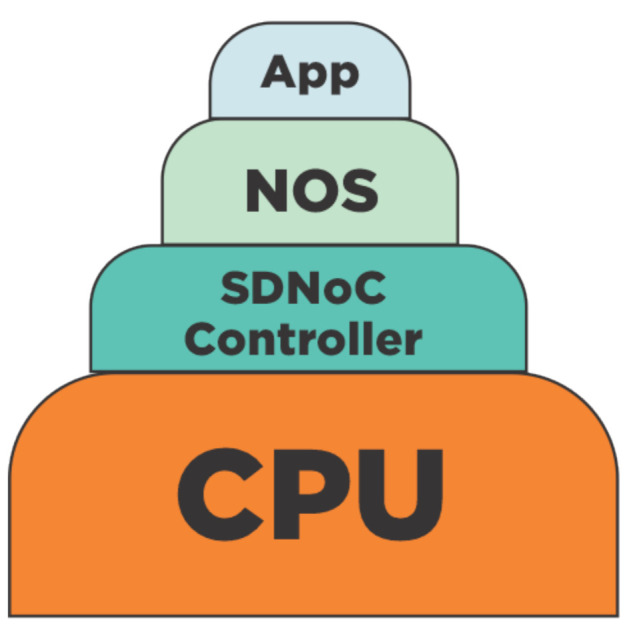
Software architecture and components in a layered SDNoC appraoch.

New designers may find complicated to understand the implementation of the SDNoC architecture. Therefore, in this section, we describe four possible implementations: Centralized and Distributed regarding the controller; and Integrated and Isolated regarding the data/control network. Before, we liked to present the software architecture for the upper layers in the SDNoC architecture, shown in Figure 4. In this illustration, the bottom layer is a central processing unit implemented in hardware with a specific instruction set architecture (ISA), such as RISC-V. Above this ISA layer, we implement the software architecture as follows: the SDNoC controller, the Network Operating System or NOS, and the applications. Each one of these software layers depends on the lower layer.

#### 4.4.1. Centralized

**Figure 5:**
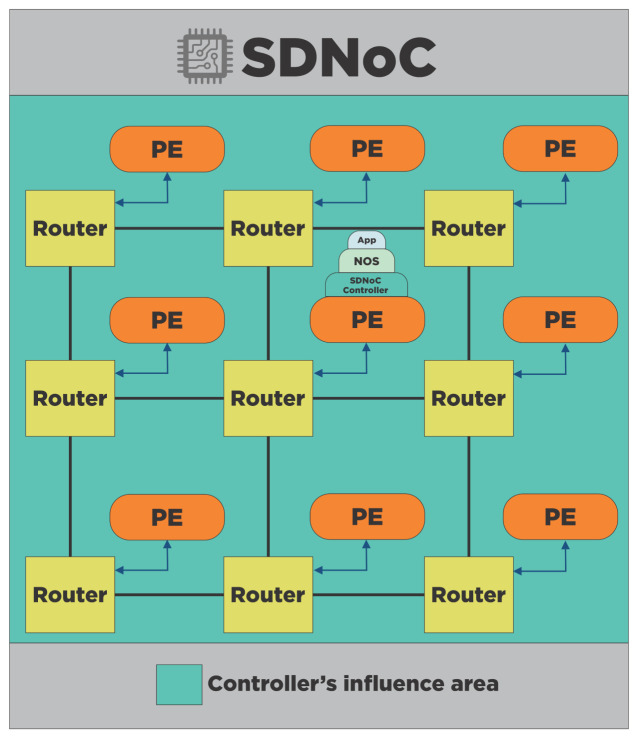
Centralized implementation of an SDNoC architecture. The illustration shows the SDNoC controller’s influence area, all the NoC, in this case. A single CPU execute all the software components.

A single CPU executes all the software components in a centralized implementation, such as the SDNoC controller, the NOS, and the applications. In Figure 5, we observe the controller’s influence area is the complete NoC, and all software components are in a single CPU (processing element at the center).

#### 4.4.2. Distributed

**Figure 6:**
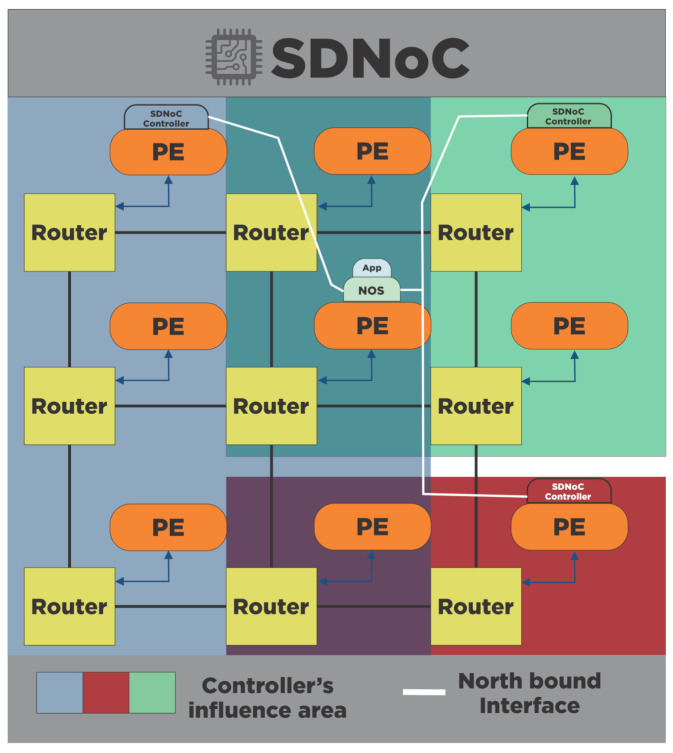
Distributed implementation of an SDNoC architecture. The illustration shows the SDNoC controllers’ influence area. The distributed SDNoC implementation spreads the software components among several processing elements in the NoC. The northbound interface, between NOS and SDNoC controller, is shown as a white line.

In the distributed SDNoC, designers spread the software components among several processing elements as Figure 6. For example, the illustration shows three SDNoC controller, each one has a different influence area, shown with different colors. The illustration present the northbound interface, shown as white lines, that interchange information between the NOS and the SDNoC controllers.

#### 4.4.3. Integrated

In an Integrated SDNoC, data and control packets use the same network. This fact generates collisions and congestion among packets, which can degrade the performance and functionality of the SDNoC. We must introduce priority forwarding to control packets to achieve a suitable latency for control packets that fulfills performance and functionality requirements.

#### 4.4.4. Isolated

In an Isolated SDNoC, data and control packets use different networks. Therefore, more resources are needed to guarantee that the control packet arrives on time at the forwarding devices to achieve its functionality and performance. The control network has lower traffic than the data network. Thus, a bus-based interconnection is a suitable option. In this type of SDNoC, we can reuse traditional interconnections to transport control packets. However, we must perform traffic analysis to ensure a fit interconnection solution.

### 4.5. Advantages and Disadvantages

Table 5 depicts a feature comparison for NoC vs SDNoC. It can be resumed as follows: SDNoC brings reconfigurability, enhances performance in terms of delay, achieves application, and traffic isolation improves security and provides network control; everything concerning traditional NoCs. However, it requires additional hardware resources in both router (table-based) and NI, and a Network Operating System is required. Then, for some simple or single application systems, this architecture is unnecessary. However, for Many-Core NoC-based systems running multiple applications, it is the right choice. If we implement the NOS in a single CPU, the computation processing required is 1/100-th of a system with 100 cores in a ten by ten 2D-mesh.

One clear disadvantage is the latency increment due to control packet transmission delay and processing. The processing latency can limit the usability of the SDNoC if the designer not considered it. Thus, an SDNoC architecture must consider control packet latency as one of the design’s critical aspects. Latency issues complicate online processing. Moreover, off-line processing is too rigid to the dynamic workloads presented by current Many-Core systems. Therefore, a hybrid approach might be a proper solution. In this approach, designers analyze several off-line use cases and obtain the fit configuration parameters to achieve functionality and performance requirements and store them for future use. Then, the SDNoC can monitor the metrics and observe if the current case seems an analyzed case. If so, it uses the stored configuration. This procedure reduces processing time for control packets but not transmission time. An Isolated network with low latency and hybrid processing could improve an SDNoC performance. However, thoughtful analysis to support these ideas is out of this paper’s scope, and we left it as future work.

## 5. Literature Review

**Figure 7:**
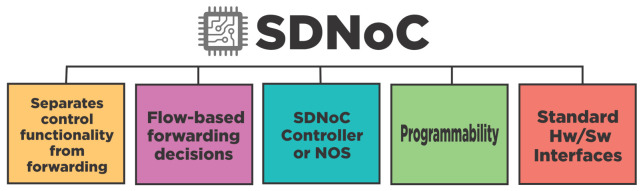
This graphic presents the NoC desired features adapted from SDN [59,63].

It is essential to recall the SDN features translated into a Software-Defined Network-on-Chip paradigm. Figure 7 shows the desired features in an SDNoC implementation. However, after the review, we will classify every surveyed work regarding the SDNoC features it has. This section presents a chronological review of SDNoC literature, grouping the works into four stages: foundations, exploration, extensions, and applications. Then a summary emphasizes the work’s contributions, gaps, and opportunity areas in SDNoC research.

### 5.1. Foundations

In this first stage, researchers adopted SDN concepts and adapted them to NoCs. Before SDN, the central controller idea was presented by Gossens et al. [65], which conjunctly with SDN inspired the SDNoC architecture. In his work, Goossens et al. presented a programmable resource reservation NoC, buffers and channels, which can embrace both central and distributed programming models. The NoC’s routers use a slot reservation table to avoid contention on a link, divide the bandwidth per link among connections, and switch data to the correct output. This slot table inspired the routing tables in the SDNoC approach. However, the paper lacks a layered architecture and a straightforward interface definition.

However, it was not until Cong et al. [66] presented the first SDNoC model in 2014. They use a simulation-based approach to test their ideas. Their proposal presented a clear separation of data path and control in the routers; however, Cong et al. built both planes within the router; a layered architecture was missing. This fact conveys that the router hardware was complicated and extensive. However, they planted the vision of an SDN paradigm into NoCs.

In the same year, Wang et al. explained a software-defined photonic NoC with a central controller [67]. Through a Photonic Network-on-Chip, they present the idea of an SDN central controller and protocol definition. They assume 15 micro-resonator rings for a photonic switch structure and a two-dimensional mesh. We can observe that the paper lacks an interface definition and a layered architecture.

In 2015, we presented a complete Software-Defined Networks-on-Chip layered architecture [20]. We describe every SDNoC layer needed to facilitate reuse and innovation in Many-Core SoCs. Despite the paper impact, the literature has omitted critical components, as we will point in the below-described works. This paper emphasizes the opportunities acquired when embracing a layered SDNoC architecture in Section 6.

### 5.2. Exploration

Once literature set the SDNoC foundations and a clear vision of their future, in the next stage, researchers and designers developed new systems and extended the SDNoC concept in and out of the chip. In 2016, we analyzed the SDNoC performance, through simulation models, regarding configuration time, throughput, and delay in several popular routing algorithms in a 2D Mesh with a central controller [47].

In [68], Scionti et al. explored an SDNoC architecture combining local ring and global 2D-mesh network to generate different logical topologies configured via software. The routers used bypassing elements to reduce latency and power-gating strategies to reduce energy consumption. They aimed to adapt the NoC logical topology to the application’s communication patterns. In order to test their models, they used simulation-based evaluations. This paper’s significant features concerning SDNoC are the configuration process incorporated into the Instruction Set of the processing elements and a switch table that describes how traffic entering a link can flow into another link. Even though they present a proper application of an SDNoC implementation, they omit the SDNoC controller implementation details. Also, the paper did not present a layered architecture with clear definitions of the interfaces. This fact limits the reuse of their work and ideas in other SDNoC designs.

Berestizshevsky explored an SDNoC architecture based on switches instead of routers, leaving behind the benefits of a packet-switched network and a flow-based routing [69]. Omnet++ simulation models serve to obtain some benchmarks results. They proposed a central network manager implemented in software and executed on a dedicated core that controls all the network’s switches using a separate control network. Despite a so-called Software-Defined Network approach, this paper lacks references to SDN ideas or previous SDNoC proposals. There is no evidence of a layered architecture and protocol or interface definitions, limiting the reuse in other designs. This paper could reveal an open architecture with an explicit programming interface to use the Network Manager for other purposes such as monitoring or energy-saving, which would improve the paper’s innovation.

Fathi et al. [43], replaced routers with switches too, to change the logical topology of the network through a central controller in an SDNoC architecture. The central controller executes the routing algorithm and makes the control decisions for all the switches. This scheme makes the controller a bottleneck in the network since it analyzes the header flit from every packet. One notable feature of this works is the first implementation of an SDNoC proposal in VHDL. However, the proposal lacks precise interface descriptions, an quantitative measurement of the hardware resources employed, and an SDNoC controller programming interface to encourage its reuse.

Ruaro et al. [51], described a layered SDNoC architecture, which uses an NoC for configuration and an SDNoC for operation. This work uses cycle-accurate RTL models and VHDL implementations to obtain performance analyzes and hardware resource estimations. Moreover, it presents a distributed controller approach for SDNoC management. This paper presents a correct implementation of some of the ideas in [20]; however, it lacks open interfaces to improve its reuse and further innovation.

Silva et al. used a single controller to evaluate the SDNoC performance regarding latency in [70]. In their proposal, the controller (manager) is implemented in software and approves the route request from every processing core in the network. They employed simulation models to obtain performance analyses. The paper omits an open programming interface that will improve its reuse and innovation.

All works explore the SDNoC approach; however, they miss an open programming interface to encourage its reuse and innovation, as we pointed before.

### 5.3. Extensions

The extensions of SDNoC to other solutions and systems begin in this stage. In 2018, Ellinidou et al. [57] extended the SDN and datacenter concepts to SoCs using an SDNoC paradigm. In this approach, several SDNoC-based SoC chips are integrated into a PCB and interconnected to create a broader network, which yields the Cloud-on-Chip concept. Moreover, they present the SoC-flow protocol, which is a secure protocol based on OpenFlow to configure the SDN switches across chips and PCBs. However, their work lacks model validation or hardware implementations.

Scionti et al. [71], proposed a scalable SDNoC to adapt the interconnections of different subsystems of a datacenter, in different chips, to deep learning applications running in the system. In this paper, the authors use routers with four local interfaces to improve performance and connectivity. Their proposal adapts the NoC topology to applications. To differentiate configuration from data packets, they use 0XFFFFFFFF as a starting flit. They use configuration functions to configure topology, router’s look-up tables, and counters. They use a Xilinx Kintek-7 FPGA to implement the SDNoC architecture. Despite an exemplary SDNoC implementation, the work presented lacks an open programming interface with enough detail to be reused and encourage further innovation.

SDNoC extensions for on-chip and out-of-chip interconnections are in this stage. Ellinidou et al., in 2019 [72], extended the SDNoC concept out of the chip to interconnect chiplets. The authors present MicroLET, a combined architecture for on-Chip and out-of-Chip networks with a clear description of the protocol and messages. They presented simulation-based model evaluations of their SDNoC architecture with XY and Odd-Even routing algorithms. However, the paper still misses an open programming interface to drive innovation and reuse.

Network Function Virtualization (NFV) is a hot topic in SDN networks. NFV introduces network functionality in virtual components, i.e., software functions running on processing elements; for example, SDN systems implement routing algorithms in software. Shantharama et al. [73] presented an extensive survey on NFV from datacenters to on-chip networks. In their work, they expressed that SDNoC facilitates the integration of interconnection systems at different scales. This paper paves the road to an integrated SDN framework from the datacenter to the chip with the same SDN philosophy.

Ibarra-Delgado et al. presented a fine-grained bandwidth control for on-chip interconnections configured in an SDNoC paradigm in [74]. The authors present the idea of a bandwidth control arbitration method for buses, switches, and routers. An SDNoC approach can exploit this method to assign the bandwidth to different applications enforcing a QoS policy at the flit level.

In this stage, the works mentioned above consolidated the SDNoC approach and extended the reach to the bus, switches, and routers, as we visioned in [20,47].

### 5.4. Applications

In this stage, new applications began to appear, and some security-focused approaches supported SDNoC architectures. Jantsch et al. presented a self-aware System-on-Chip proposal to manage and report their system behavior [39]. The authors present a framework to implement different resource allocations, process monitors, and a learning framework with the recollected statistics that optimize the systems’ performance. Despite not using an SDNoC, we believe that an SDNoC could help achieve its goals by generating a modular solution in different SDNoC architecture layers. For example, a monitoring solution at the control layer working with a learning solution at the network orchestration functions in the management layer.

In 2018, Temuçin and Imre presented a Software-Defined Photonic Network-on-Chip [49]. They aimed to centralize a contention-free and conflict-free scheduling algorithm to solve the routing and wavelength assignment in optical networks. The controller has predefined schedules of different traffic patterns to improve the speed and reduce the implementation complexity. In the same way as other papers, this work did not present a layered architecture or a programming interface to encourage its reuse or innovation.

Security is one main topic in this stage. Ellinidou et al. [64] extend his previous work and addressed the secure communication problem for SDNoC router configurations in their Could-on-Chip architecture. They described a secure protocol to manage SDNoC routers, including three phases, a private key derivation phase, a group key agreement (GKA) phase, and a data exchange phase. This protocol ensures that basic security primitives are preserved and provide secure communication. The paper presents evaluations of Sharma and TENG, both GKA protocols based on Mininet simulations. The paper’s main drawback is the lack of well-defined interfaces to encourage the implementation of their secure protocol in other designs.

In [17], Rouaro et al. presented the idea of achieving SoC security through an SDNoC approach. They test their proposal of a secure SDNoC framework with Denial of Service, Flooding, and Spoofing attacks using SystemC models and VHDL implementations to evaluate additional hardware resources. They based their baseline architecture on their previous paper [51] and improved the Network Interfaces with a Secure Configuration Logic (NI-SCL). This element introduces a secure protocol for network configuration. The lack of open interfaces to exploit this framework on other designs is a detriment.

Then, in [16] Ruaro et al. defined secure zones in an SoC employing an SDNoC architecture. They use an application admission mechanism to accept new applications in a secure zone. Therefore, secure applications are isolated physically using the SDNoC approach. The authors used SystemC simulation and VHDL for the synthesis to evaluate their proposal. This paper presents an explicit use of an SDNoC architecture for security application admission in NoC-based SoCs. Nevertheless, the paper did not present an open programming interface to encourage its reuse in other designs.

### 5.5. Summary

**Figure 8:**
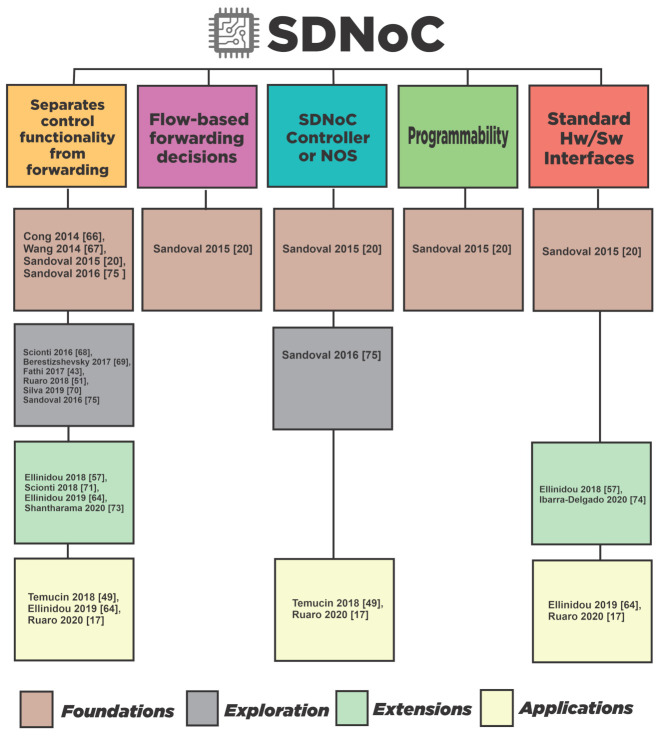
Literature classification regarding the SDNoC desired features (columns) and chronological stages (colors at the bottom).

Figure 8 presents the summary of the survey results, complementing Figure 7, and classifying the literature by the SDN features that display. The SDNoC approach has five features; however, as we can observe in Figure 8, researchers concentrated their efforts on three features, control plane separation from forwarding, a central controller, and interfaces. However, they have left out flow-based decisions, programmability, and standard interfaces. We can see the literature gaps In Figure 8; thus, we propose redirecting the research endeavors to fill these gaps.

Further research efforts need to consider deploying SDNoC features, such as detailed API implementation and standardization between the control plane and the data forwarding plane. Some other SDN characteristics are missing from the literature, such as flows as the unit of forwarding decisions; software abstractions with a global view of the network; a Network Operating System; network programmability; and well-defined and standard interfaces to enhance the reuse of SDNoC solutions in other systems design. In this paper, we propose to research these issues further.

## 6. Challenges and Opportunities

In this section, we discuss some of the challenges and opportunities detected in the SDNoC literature. We explain the benefit that each one will convey.

### 6.1. Challenges

We classified the challenges regarding their implementation in hardware, middleware, and software. Also, we briefly introduce each concept to illustrate the impact of the component in the architecture.

#### 6.1.1. Hardware

Hardware implementations depend on the process technology (silicon, photonic, or 3D), platform (ASIC, FPGA, or hybrid), and application (Radiation-hardening, electromagnetic shielding, industrial environment, and operating temperature) requirements.

- SDNoC elements: In a Many-Core paradigm, where the NoC scales to thousands of network elements, we need to develop hardware implementations of buses, switches, routers, and network interfaces employing few resources and low energy consumption.
- Programmable devices: The Software-Defined approach requires programmable underlying devices. One of the main goals is to achieve a reconfigurable topology, physical or logical, that adapts to the application’s data dependency requirements. A hybrid or dual packet/circuit-switched implementation would help service differentiation to achieve QoS requirements.
  - -Network Interfaces: We need to adapt (program) the packet size, flow, memory addresses, and network address. Moreover, a traffic regulator approach would help to characterize traffic before entering the network, for security and performance goals.
  - Buses: Dynamic reconfiguration buses with programmable priorities and arbitration policies to ensure bandwidth allocation [74], QoS, and resource reservation.
  - Switches: We can use port reconfiguration and dynamic arbitration policies to ensure QoS to real-time processes.
  - Routers: path (route) reconfiguration is a desirable feature and a must in SDN.
- Wireless SDNoC routers: Wireless NoCs bring connectivity enhance that an SDN approach can easily exploit.
- Introduce SDNoC architecture with new process technologies: Optical, wireless, and 3D, to cope with thermal and latency issues.
- SDNoC for Inter-Chip networks: Off-Chip interfaces adapted to on-Chip Networks to interconnect several chips in a PCB or larger designs as presented in [64].

#### 6.1.2. Middleware

These software components offer services aggregated as needed that complement the network operating system, such as secure protocols, network protocols, and configuration processes. We can characterize them by function (configuration, monitoring, or information gathering), device (routers, switches, buses, network interfaces, controller-to-controller), or generic drivers for network elements. Also, they may provide an open programming interface, packets, and protocol definitions.

- Secure protocols: As Ruaro et al. [17] propose, a secure protocol to configure the SDNoC elements is needed. Moreover, we need to adapt the secure protocol to different implementations and network scales.
- Network protocols: SDNoC design will need new protocols for network information exchange, operation status, configuration, and further. For example, a DNS-like service to find network addresses based on memory addresses.
  - Addresses-directory service: In an SDNoC-based Many-Core platform, we will need to translate memory addresses from the memory space to network addresses. This process is simple in a static environment; however, dynamic workloads may change this memory mapping at runtime. Therefore, an address-directory service will help the dynamic reconfiguration.
  - Address-allocation service: Address allocation may change in a dynamic environment; therefore, the system will need an address-allocation process for new applications or services or reallocated unused address spaces. Furthermore, we will create virtual networks or sub-networks in the system to isolate traffic for performance or security reasons. This example would lead to achieving autonomous systems-like designations into on-Chip networks with different routing algorithms and policies.
  - Location-based services: We can exploit location information to treat packets in a differentiated manner, establish secure zones, or location-based forwarding device configuration (routers, switches, buses, and network interfaces). For example, a network interface may be in an energy-saving zone and adapt the packet size to reduce transistor switching and energy consumption.
- Hypervisors: These components derive a partial or global network view with the information gathered from the forwarding devices. With this view, the component presents a software abstraction of the network to upper layers. For example, a view of memory-only, CPU-only, or IP Core-only networks with every application’s resource allocation in the system.
- Network Function Virtualization: As in SDN in networking, we can virtualize some network functions such as routing, filtering, and access lists. However, the challenge is to achieve a low latency software implementation.

#### 6.1.3. Software

The management framework that we conceive has several software components such as Network Operating Systems, network drivers, network applications, network optimization frameworks, and network orchestration functions, to mention a few.

- Network drivers allow having specialized functions and software abstractions/models of some parts of the network such as routers, secure zones, applications, and virtual networks. These components present a programming interface to use or reconfigure some parts of the network without knowing the low-level implementation details.
- The Network optimization framework allows solving complex optimization problems with different goals and constraints. As we mentioned above, the future and current NoCs require several dynamic network optimizations such as routing, mapping, topology, and scheduling. The SDNoC architecture requires software components to execute several optimizations dynamically with goals and constraints translated in the network configurations for lower layers.
- The Network applications are functions to optimize the network or obtain information from the network, such as an energy-saving function for routing or scheduling. Furthermore, a desirable feature of SDNoC is observing the buffers, energy consumption, link use, and temperature executed by a monitor application.
- The Network orchestration functions manage conflicts among different optimization functions or goals, with the plan of a greater goal or knowledge. As optimization goals have conflicts, we need policies to resolve these issues, achieve adequate system performance, and assure QoS requirements.
- The Network Operating Systems englobes all the features above in a single and well-articulated software component. Furthermore, different systems will need different Network Operating Systems specialized in some areas such as energy-saving, thermal management, real-time performance, multimedia processing, or artificial intelligence, to mention a few.

### 6.2. Opportunities

When we review the SDNoC literature, we observed some opportunities to thrive the SDNoC research.

- Extend the Instruction Set of some microprocessor architectures –such as RISC-V– to include networking functions or instructions for sending messages through the network.
- Include network sensors to monitor within a machine learning adaptive architecture. Some physical variables of interest are temperature, electromagnetic field, voltage, electrical current consumption, optical power, signal-to-noise ratio, error rate, error correction rate, and channel/link state.
- Encourage open hardware/software/architecture initiative to encourage innovation with clear and standard programming interfaces to drive HW/SW component reuse.
- A flow-based management of routing functions is a cornerstone of the SDN missing from the SDNoC literature.
- A programmable network is a clear opportunity for SDNoC. This feature will dynamically adapt the network resources to the application currently running in the system.
- A network programming language for SDNoC will improve adoption, standardization, and new features.

## 7. Conclusions and Perspectives

The complexity of NoC management problems associated with Many-Core SoCs implies a different solution approach than traditional NoCs. The layered SDNoC architecture presents a modular strategy where the separation of concerns permits innovation at different abstraction levels. Despite the high-grade quality of the works in the SDNoC literature, some SDN features are missing. This fact conveys that solutions to management problems are not reusable in new designs, increasing the development and time to market. Therefore, SoC architects refuse to employ an SDNoC architecture for their future designs.

NoC management includes challenging optimization problems; in an SDNoC architecture, researchers can focus on these problems without coping with the low-level details. Furthermore, we observe that an optimization framework for Many-Core systems is a required component.

This paper surveyed the literature regarding SDNoC and observed some challenges and opportunities to improve the state-of-the-art. Once we, the research community, overcome the challenges, we will facilitate SDNoC implementations and spread their use in new SoC designs. The most important opportunity is to impulse an open architecture where researchers can apport their contributions and new designs use them with easy integration and configuration.

## Figures and Tables

**Table 1:** Large Silicon Valley companies bought NoCs companies. Qualcomm made a partial purchase on Arteris, which preserves its patents.

Company	Buyer	Date
Arteris (partial)	Qualcomm	2013 [1]
Netspeed	Intel	2016 [2]
Sonics	Facebook	2019 [3]

**Table 2:** Recent chips by relevant companies. We describe the type of cores and the on-Chip interconnection. All designs have a Many-Core approach.

Chip	Company	Features(Cores)	NoC	References	Year
Xeon Phi	Intel	72 Xeon cores	Ring (Photonic) Knight’s Ferry-Corner/2D Mesh Knight’s landing	[6]	2016
OpenPiton + Ariane	-	65,536	2D Mesh crossbar	[4]	2019
DaVinci Ascend910	Huawei	32 DaVinci cores	Uses Arteris NoC Ring and Mesh bus	[7]	2019
TrueNorth	IBM	4096 Neurosynaptic cores	2D Mesh	[8,9]	2019
Cloud A100	Qualcomm	16 Neural processors	Arteris	[1,10]	2020
Loihi	Intel	128 Neuromorphic cores	A 2D Mesh NoC with up to 4096 on-Chip cores	[11]	2020
Epiphany-V	Adapteva	1024 RISC-V cores	Three NoCs	[5]	2020
AI Processor	-	16,384 (128 × 128) Nano cores	7 NoCs	[12]	2020
Versal (ACAP)	Xilinx	-	The NoC topology is not regular defined by the design	[13,14,15]	2020

**Table 3:** Chip faults exhibit different syndromes and have various causes that affect NoC reliability [18,25,26,27].

Type of Fault	Syndrome	Characteristics	Cause	Solution
Transient	One or more bit-errors in a transmitted packet	Random and short duration	Crosstalk effect or alpha or neutron particle strikes	Error checking/correcting schemes or retransmissions
Permanent	Faulty elements	Non-recoverable device defects	Due to manufacturing defects or device wear-out caused by Negative Bias Temperature Instability (NBTI), Hot Carrier Injection (HCI), Time-Dependent Dielectric Breakdown (TDDB)	Redundant resources available to replace the faulty components

**Table 4:** Recent literature classification of NoC optimization problems and goals. These works could benefit from an SDNoC architecture. SDNoC will provide a reuse framework for the different solutions proposed in every paper into a single SDNoC system.

Problem/Goal	Thermal Aware	Latency	Performance (Throughput)	Power Efficiency	Fault Tolerance	Security
**Mapping**	[29]	[40,41]	[40,42]	[26,27,31,36,40,41]	[25,26,27,29]	
**Topology**				[36,37,43]		
**Routing**	[44,45,46]	[44,45,47,48]		[48]	[38]	[34]
**Scheduling**	[29]		[49]	[26,27]	[27]	[34]
**Management**	[19,28,30]	[32,50,51]	[50]	[30,32,50,52,53,54]	[18,32,50,55]	[17,33,50,56,57]

**Table 5:** Feature comparison for NoC versus SDNoC.

Feature	NoC	SDNoC
Reconfigurability	None	Routing, arbitration priorities
Routing Algorithm	Fixed for all flows	Flow adaptive
Hardware	Simple	Additional complexity
Delay	Fair	Lower
Applications	Mixed	Isolated by traffic routes
Security	None	Flow traffic pattern assurance
Traffic	Mixed	Flow or application isolated
NOS	None	Present
Router	Routing algorithm	Table-based
NI	Simple shell	Additional configuration hardware
Network Control	None	Centralized or distributed
